# “*I know who I am*”: Gender-creative children Draw-and-Tell their social and healthcare experiences

**DOI:** 10.1080/29968992.2026.2653541

**Published:** 2026-04-05

**Authors:** Eline L. Lenne, Ben Anderson-Nathe, Christina J. Sun, Martha Driessnack

**Affiliations:** a Faculty of Social and Behavioural Sciences, University of Amsterdam, Amsterdam, Netherlands;; b School of Social Work, Portland State University, Portland, OR, USA;; c Anschutz Medical Campus, University of Colorado, Aurora, CO, USA;; d School of Nursing, Oregon Health and Science University, Portland, OR, USA

**Keywords:** Gender-creative, Draw-and-Tell conversations, arts-based research, pediatric healthcare, social support

## Abstract

Gender-creative children have an elevated risk of healthcare avoidance and adverse health outcomes related to stigma and discrimination. Despite evidence that family affirmation is protective and pediatric providers influence care outcomes, young children’s perspectives related to their social support and clinical experiences remain underrepresented in health research. Indeed, children’s agency has been historically restricted in healthcare and health research. This community-engaged, artsbased, qualitative study used Draw-and-Tell Conversations—a participatory approach combining drawing, storytelling, and writing—with 12 U.S.-based gender-creative children (ages 5–10). Although children’s caregivers completed surveys to contextualize findings, the project highlighted children’s capacity to report on their own experiences. Data were analyzed using reflexive thematic analysis and poetic inquiry. Participants expressed discomfort in clinical settings but identified trusted adults with whom they could discuss gender. Drawing facilitated confidence and clarity in communication. Findings reveal missed opportunities in pediatric care and research to engage directly with gender-creative children. This study contributes child-centered, empirically grounded recommendations for gender-affirming care and highlights the value of arts-based methods in health research with children.

## Introduction

Over the past decade, the numbers of transgender and gender-creative children have risen, reflecting increased social recognition and affirmation of gender diversity. And yet, their voices and perspectives continue to be largely absent from the literature and clinical care settings (Horton, 2023, 2024). Approximately 1.3–2.7% of children in the US identify as transgender, gender-creative, or otherwise gender-expansive (Lillemoe et al., 2023).^1^ Across the United States (US), transgender people are being threatened by active, coordinated efforts to erase them from public life, medical care, legal protections, and from history itself (Bravo, 2025; Marcus, 2025; McNamara et al., 2024). Gender-creative children face higher rates of stigma, social isolation, and low self-esteem resulting in adverse health outcomes including mental health concerns and emergency department admissions (Katz-Wise et al., 2022; Lillemoe et al., 2023; Rafferty, 2018). Further, they are three to five times more likely to attempt suicide than their age-matched cisgender and heterosexual peers (Chang et al., 2022; Di Giacomo et al., 2018; Schultz et al., 2022). Due to this stigma and discrimination, health care avoidance often carries over into adulthood, further contributing to poor physical and mental health outcomes for transgender individuals (Kcomt et al., 2020). Transgender adults are more likely to experience chronic illnesses (e.g., cancer and heart disease) resulting from missed preventative care and screening visits due to discrimination-related health care avoidance (Rich et al., 2020).

Pediatric primary care providers (PCPs) are often gender-creative children’s first point of contact in the healthcare system (Allen et al., 2019; Rafferty, 2018; Weiselberg et al., 2019). However, they may lack LGBTQ-specific knowledge, resulting in inadequate care and insufficient guidance for children and their families (Kitts, 2010; Lena et al., 2002; Riley et al., 2011; Shires et al., 2018). The initial interactions gender-creative children and their families have with pediatric providers may affect how children are affirmed or denied in their gender identity at home, school, and in their communities, which may in turn influence long-term physical and mental health (Durwood et al., 2021; Ehrensaft, 2016; Ehrensaft et al., 2018; Rafferty, 2018). When primary caregivers and families are well-supported by pediatric PCPs, they can better support their child’s long-term wellbeing (Fussell, 2011). Conversely, pediatric PCPs can miss opportunities to positively impact caregiver-child relationships when gender-affirming care is overlooked in the pediatric healthcare context.

In research and clinical practice, school-aged gender-creative children are routinely overlooked based on an assumption that they cannot yet express themselves in useful ways and do not yet need gender-affirming care (Ehrensaft et al., 2018; Gill-Peterson, 2018). To date, the literature primarily focuses on medical guidelines and implications related to pubescent gender-creative adolescent and adult medical transitions (de Vries et al., 2014; Horton, 2023, 2024; Katz-Wise et al., 2022; Olezeski et al., 2020). Studies related to school-aged children typically rely on caregiver proxy or near-peer reports, in which adolescents and young adults reflect on their earlier childhood experiences (Horton, 2022, 2023, 2024; Lesser, 1999; Medico et al., 2020; Rahilly, 2015; Steensma et al., 2011, 2013a, 2013b). Of the studies that do engage prepubescent children directly, few offer the individual perspectives that emerge from the use of developmentally sensitive approaches to qualitative data collection and analysis. Instead, studies have relied on quantitative measures, such as depression and anxiety scales (Durwood et al., 2017; Olson et al., 2015; Olson et al., 2016). Given the dearth of research pertaining to *pre*pubescent gender-creative children’s qualitative perspectives, inviting gender-creative children to offer their experiences and insights may help providers, researchers, and caregivers identify where and how to bolster approaches to care. Listening to these children, in contexts where their voices are often quieted in favor of adult perspectives, may uncover novel approaches to better support them (Davies et al., 2024; Lenne et al., 2023).

To frame this work, we drew from poststructuralist, transgender, and queer theories. These theories share a commitment to challenging normative categories and disrupting fixed meanings (Hesse-Biber, 2017). Poststructuralism aims to interrogate the instability of meaning and emphasizes the potential for reinterpretation and re-signification, both exposing and unraveling dominant narratives (Belsey, 2022). Transness exemplifies this disruption, troubling the assumption that sexed bodies must correspond to binary gender identities (Butler, 1993, 2024). From a poststructuralist lens, transness destabilizes the presumed coherence between physical form and gendered meaning—between embodiment and identification, between biology and performance, between the signifier and what it is presumed to signify.

Queer theory emerged in the early 1990s from feminist theory, post-structuralism, and LGBTQ+ activism, and challenges the notion that gender and sexuality are natural or fixed categories (Jagose, 1996). Transgender theory gained prominence in the mid-1990s and early 2000s through the work of transgender scholars and activists who critiqued both cisnormative frameworks and earlier feminist and queer theories that had often marginalized or pathologized trans experiences and minimized the embodied and material consequences of gender transgression (Stryker, 1994, 2006). Central to these theories are the concepts of heteronormativity, which is the assumption that heterosexuality is the default or ‘normal’ sexual orientation, and cisnormativity, which presumes that people’s gender identities naturally align with the sex they were assigned at birth (Warner, 1991). By exposing these norms as socially constructed rather than inevitable, queer and trans theories create space for recognizing the diverse ways people experience and express gender and sexuality. Both queer and trans theories offer critical, interdisciplinary approaches that seek justice for those marginalized by dominant constructions of gender and sexuality (Hesse-Biber, 2017; Jagose, 1996). These perspectives resist essentialist understandings of identity, instead emphasizing multiplicity, context, and fluidity. While queer and trans theories differ—particularly in how they approach the experiences and politics of transition—they are aligned in their shared critique of cisnormativity, heteronormativity, and rigid gender binaries (Butler, 1990, 2024; Love, 2014).

### The current study

We used a community-engaged, arts-based approach to understand gender-creative children’s experiences with gender, health, and social support. Our research questions were:

- What would help gender-creative children feel safe to express thoughts, feelings, and experiences related to gender?
- Who do gender-creative children go to when they want to talk about their health and/or gender, and why?
- What is it like for gender-creative children to attend pediatric healthcare visits?

## Methods

In this qualitative, arts-based study with prepubescent gender creative children, we sought to listen to, amplify, and support gender-creative children. Arts-based methods are particularly generative for engaging with queer, transgender, and poststructuralist theories, as they center fluidity, embodiment, and resistance to rigid epistemological boundaries (Denton & Cain, 2023). These methods also disrupt normative modes of scientific knowledge production by privileging affect, movement, and multiplicity in meaning-making, ensuring that seldom-heard groups such as younger children are engaged and empowered in research (Denton & Cain, 2023; Nathan et al., 2023).

### Positionality

In the interest of positioning ourselves in this work, three of us identify as white and one as Asian-American. Two of us identify as queer. While all the authors identify as cisgender or cisgender-passing, we all have personal and professional experiences caring for and supporting gender-creative children and the broader transgender community. Our experiences, along with those of our community partners with whom we regularly consulted, helped shape this work.

### Setting and Sample

#### Sample

We used convenience and snowball sampling to recruit 12 gender-creative prepubescent children from two metropolitan areas in the US Pacific Northwest and Northeast, where the first author could conduct in-person interviews. Participants included gender-creative children between the ages of 5 and 10 who had undergone no medical interventions related to their gender identity. Drawing and conversational components were conducted in English.

#### Procedure

Eligible participants (child-caregiver dyads) reviewed a packet with information about the study, including consent forms (primary caregiver permission and child assent) and a social-story booklet (Te One, 2011) designed in consultation with gender-creative children, which described the research project and process for children to review. Efforts to maintain and strengthen children’s sense of agency were incorporated throughout the research session (e.g., children had choices about research materials, depiction of subject matter, whether caregiver was present, whether to share drawings with the public, and the type of gift card they wished to receive).

Half of the participants chose to meet at home, and the other half opted for public indoor and outdoor spaces (i.e., parks, academic conference room). The sessions lasted an average of 50 min, ranging from 34 to 95 min. The sessions included time to build rapport and acclimate to the setting and project. The Portland State University Institutional Review Board approved the study and provided ongoing oversight (HRPP #238255–18). All child participants provided written assent and caregivers provided written consent prior to enrollment in the study.

### Data collection

We used an arts-based approach to data collection to ensure that our data were dynamic, creative, and aligned with our epistemological and theoretical stances, while also complementing the study participants’ way of being (Nathan et al., 2023). To ensure that children were meaningfully engaged and centered in the research, we used the Draw-and-Tell Conversations method (DTC; Barfield & Driessnack, 2018; Coyne et al., 2021; Driessnack, 2006; Driessnack & Gallo, 2013; Kim, 2023; Linder et al., 2018; Pope et al., 2019; Robledo Castro et al., 2023; Water et al., 2020; Wiseman et al., 2019). The DTC method affirms children’s agency and offers them a chance to formulate thoughts visually prior to verbalizing them, leading to richer conversations and data. Children had access to watercolor paper and various art materials including watercolors, pens, colored pencils, graphite pencils, and beeswax crayons. In some cases, children preferred to build with Legos or take a walk in the woods in lieu of drawing. We invited children to draw or construct two pictures—one depicting the most important people in their lives and one depicting a healthcare encounter—and discussed each picture after they finished drawing using an open-ended interview guide. While children drew, we invited caregivers to complete a Qualtrics (March 2024 version) survey containing questions about their demographics and healthcare experiences to contextualize findings.

### Analytic design

We used multiple methods of analysis to process the various data we collected (i.e., recordings, drawings, caregiver surveys). The combination of analytic methods enabled us to build connections between and across data, keeping children’s perspectives in focus throughout. We hand-transcribed interviews as a form of data immersion, listening for children’s changes in intonation, pauses, and negotiations with their caregivers related to the telling of their stories (Riessman, 1993). We noted body language, intonation, and side-conversations with caregivers in the transcripts and in some cases reflected further on these subtle exchanges in memos.

#### Reflexive thematic analysis

We conducted a reflexive thematic analysis of the transcripts on Dedoose (Version 10), using a hybrid deductive and inductive approach at a semantic level (Braun & Clarke, 2021), because we recognize that meaning can be found not only in patterns in data but also through interpretive frames incorporated into the analysis. We generated themes through an iterative process of reading and re-reading transcripts, coupled with a review of observational memos and reflexive journals. Reflexive thematic analysis aligns with post-structuralist perspectives in its rejection of positivist notions of objectivity and its emphasis on meaning as socially constructed, fluid, and context-dependent (Braun & Clarke, 2021). We did not seek to obscure our role as researchers, rather we acknowledge that we actively shaped the analysis and embrace our interpretations as dynamic, reflexive, and contextually-specific (Braun & Clarke, 2021).

#### Poetic inquiry

We deepened our engagement with the data using I-Poetry to embrace the complexity of the participants’ lived experiences and the uncertainty, subjectivity, and ongoing production of knowledge (Nicolaou & Eloff, 2024; Richardson & St. Pierre, 2005). We identified all first-person I-statements and extracted these phrases from each of the twelve transcripts to compile them into a spreadsheet. We deleted peripheral or repeated statements (e.g., ‘I don’t know’). The mean number of I-statements shared per participant was 10.73, with a minimum of 6 and a maximum of 19. We reviewed the I-statements and grouped them by thematic content. From there, we created I-Poems, capturing participants’ experiences across several themes in an aggregated format. Every child’s voice was reflected in at least one of the I-Poems and, in many cases, they can be heard across all five poems.

The process of creating the I-Poems was iterative. We organized the participants’ I-statements to create a poetic flow. As we constructed the poems, we referenced preliminary findings from the reflexive thematic analysis to clarify our interpretations. We revisited the poems over the course of several weeks, alongside reviews of the transcripts and memos, and then revised them to correspond with five unique themes. We included discursive brackets to highlight statements that follow discourse markers or fillers, such as ‘you know,’ ‘like,’ and ‘um’: *‘I would say [like] what’s your gender or something before I tell them.’* The statements that follow such fillers often convey profound ideas that may require additional time to express verbally (Laserna et al., 2014).

#### Visual content and descriptive analyses

We inventoried the visual content depicted within the children’s drawings to identify patterns and used descriptive statistics to analyze the caregiver survey data. Relevant demographic data are shared in aggregate form to protect the participants’ identities.

#### Member check

After finishing our analysis, we put together a booklet containing the children’s drawings and the I-poems and sent the booklets to participants along with an invitation to reflect on the findings. Four of the twelve participants offered their reflections via voice memos and they all shared that the poems and drawings felt relatable and mirrored their own experiences.

## Results

### Participants

Twelve children ages 5- to 10-years-old participated in the study, with a mean age of 7 and median age of 6 (SD = 1.9). Five children (41.7%) identified as nonbinary, four (33.3%) identified as trans female, one (8.3%) identified as trans femme and non-binary, one (8.3%) identified as trans male, and one (8.3%) identified as male. Two children identified as mixed-race (16.7%), while the remainder identified as white and non-Hispanic. All participants had at least one caregiver with a graduate-level education (Master’s or Doctoral degree). Most caregivers (*n* = 10; 83.3%) identified as cisgender, while two identified as gender-creative. Eight interviews were conducted in the Pacific Northwest, and four in the Northeast. Pseudonyms and participants’ chosen pronouns are used in all excerpts.

### Themes

Each of the five themes is introduced by an I-Poem and the titles include excerpts from children’s narratives. The five themes are: (1) ‘*The sun means my family is warm and loving*’: Sources of Social Support; (2) ‘*I have a cat*’: Animals as Social Support; (3) ‘*I make those in my imagination*’: Windows into Children’s Lifeworlds; (4) ‘*I just want to tell you*’: Talking and Not Talking About Gender; and (5) ‘*I just take deep breaths*’: Navigating Clinical Experiences.

#### Theme 1: ‘*The sun means that my family is warm and loving*’: Sources of Social Support

I made my picture.

So, here’s my mom, and this is my sister. She has curly hair. And then this is my dad.

I made daddy with long hair, let’s just pretend he’s a teenager.

I trust everyone in this picture.

I feel comfortable and safe with everyone here.

I drew me ‘cause I care about me.

I like when my mom is close by.

I drew her next to the sun. The sun means that my family is warm and loving.

I get to see them every day.

I get to live with them. They care about me.

I drew a mystery person with no arms. I like playing with her.

I want to whisper something to you, mom.

**Figure 1.**
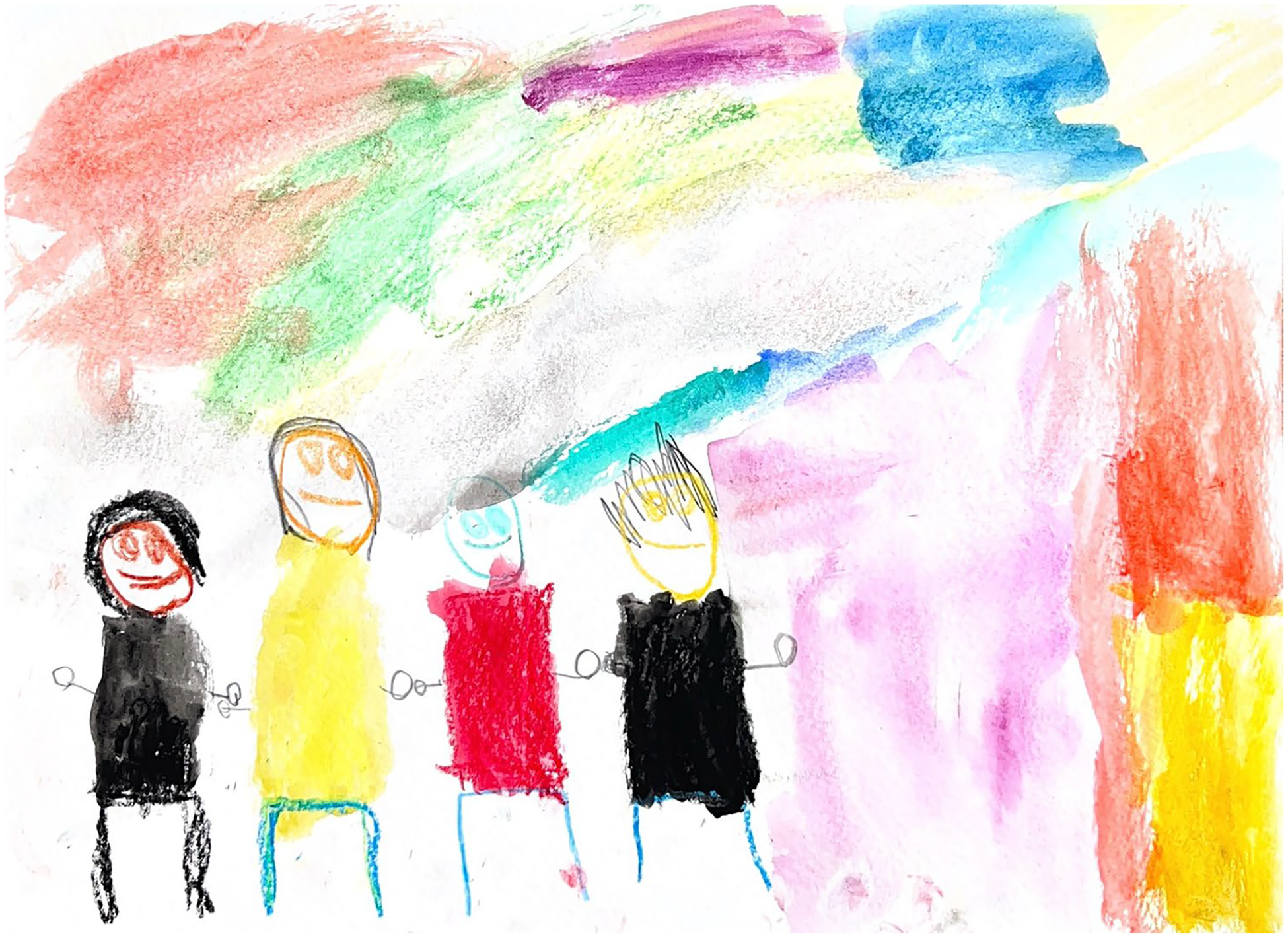
Noa (5) drew themselves with their parents and big sibling.

Family and close friends were prominently featured in most (*n* = 10/12; 83.3%) of the children’s stories. Children described how their families were always there for them, helped them do things, and supported them when they needed it. Kai shared, ‘*My mom is important in my life because she takes care of me with my dad and plays with me and gives me breakfast. And [my sister] is important because she plays with me a lot*.’ According to Mila, what makes her family special is ‘*That I get to see them every day. That I get to live with them. That they care about me*.’ Charlie, who lives in a multigenerational home, shared that he felt comfortable talking to his mother and grandmother, ‘*Mama and Lita*,’ about gender. Some children felt comfortable talking to their siblings or close friends about gender, and some did not. June, whose sibling is also gender-creative, shared that they felt comfortable confiding in them because of their shared experiences (Figure 1).

**Figure 2.**
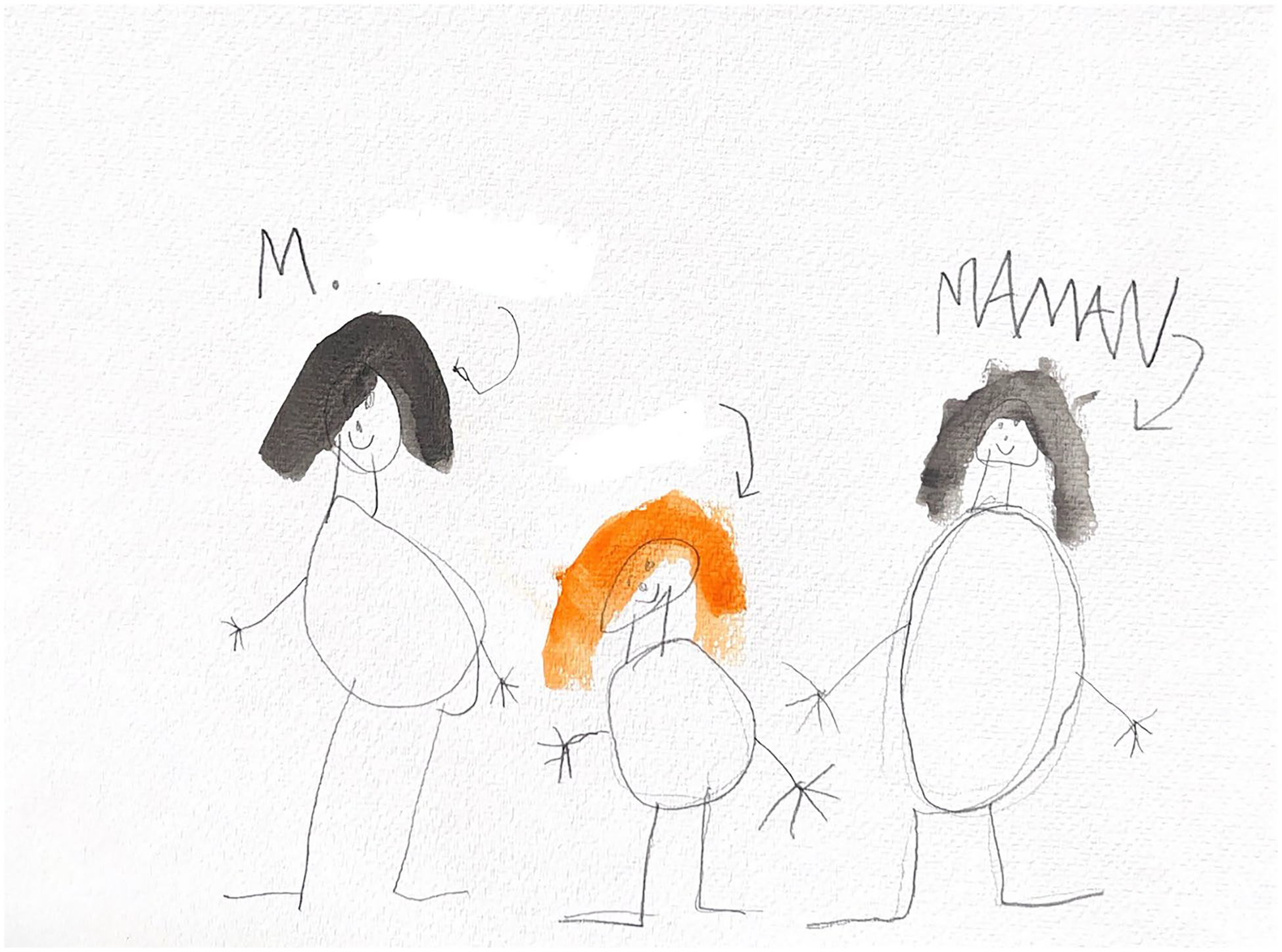
Kai (7) drew their teacher, sibling, and mother.

Four children (33.3%) drew and/or talked about their teachers, describing them as trusted adults they could confide in. A few described instances in which their teachers stood up for them when they felt unsafe or experienced bullying related to their gender identities. Mila shared, ‘*She knows I’m trans. We don’t really talk about it much because she already knows I’m trans […] she still respects me, just doesn’t want to talk about it because a lot of kids are always around at school.’* Because Mila’s teacher knew about her gender identity, Mila felt she could safely confide in her teacher when she encountered bullying. Many of the children expressed feeling a depth of connection with their teachers that facilitated self-confidence and trust (Figure 2).

Notably, the children did not name other adult professionals with whom they felt comfortable having conversations about their health and gender, and two explicitly stated that they would ‘*never*’ talk to their healthcare provider about gender.

#### Theme 2: ‘*I have a cat’*: Animals as social support

I’m hoping that I’m going to have enough money to buy myself my own pet. Hopefully a bunny!

Hopefully a bunny.

I have two bunnies, three cats, and one snake.

I talk to my neighbors’ goats, and chickens, and bunnies.

I trust Francine and Olive the most. Francine is my dog and Olive is my bunny.

I have three mice. One of them is named Milly. One of them is named Daisy. And one of them is named Wren.

I holded her and saw that her eyes were blue.

I have a cat named Rumi, but she’s very grumpy with me.

**Figure 3.**
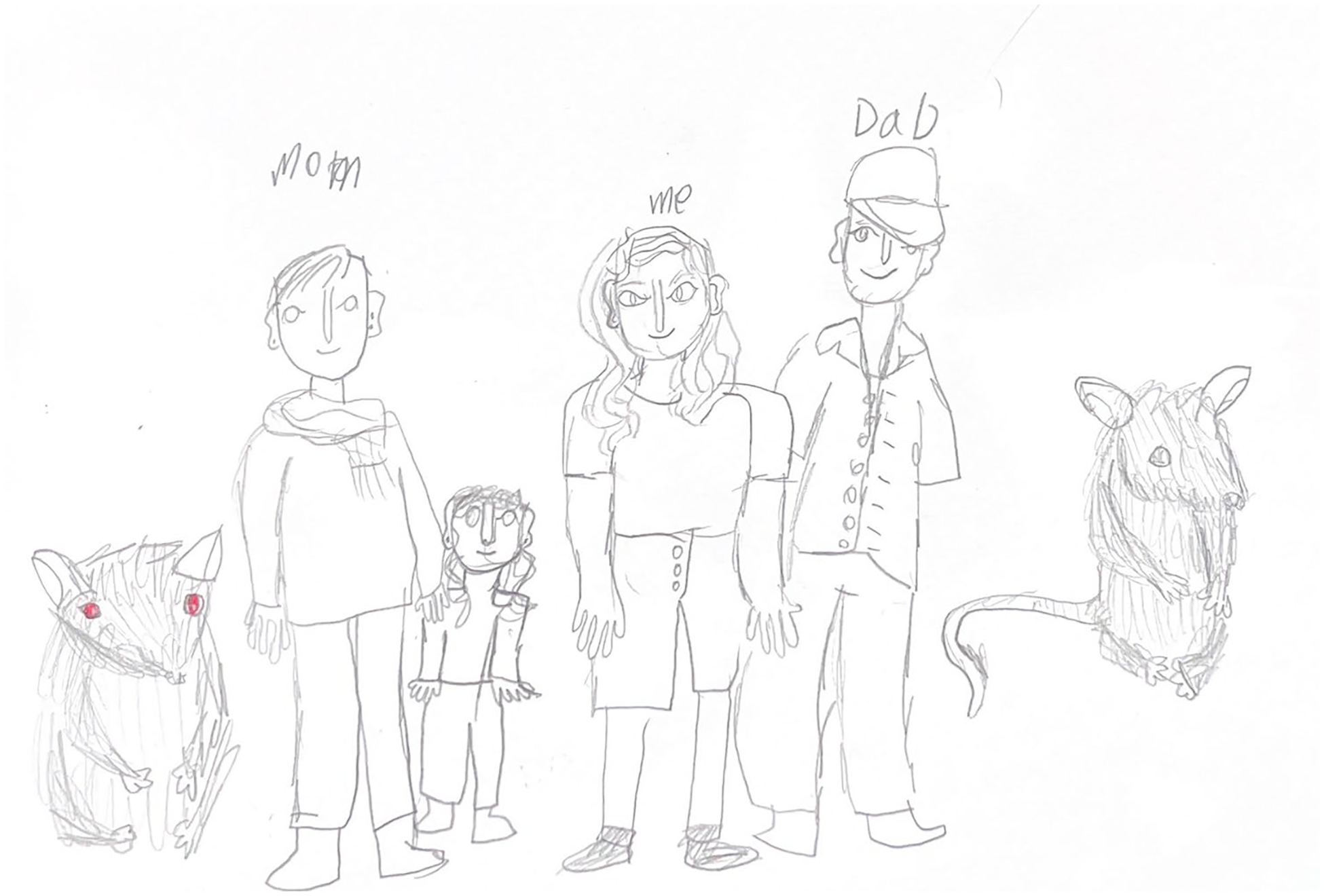
June (9) drew their self-portrait alongside their parents, sibling, and pet mice.

Although we did not directly ask children about animals, a majority (*n* = 8/12; 67.7%) were eager to draw and/or talk about the animals in their lives. During the interviews, we met several pets and stuffed animals. In cases where pets were not present during the interviews, children begged their caregivers to share pictures on their phones (Figure 3).

When the children talked about animals, they visibly relaxed. The topic offered an opening– the children perked up and shared with excitement and fondness. Mariel, in response to a question about her family, shouted, *‘We have three pets!’* When we asked Rain to share more about the important people in her life, she asked, ‘*Can they also be animals?*’ In another interview, Noa responded as their alter-ego, Apples the cat, and talked about visiting the veterinarian instead of the pediatric PCP. We asked Noa what the veterinarian knows about Apples and Noa responded, *‘That she’s a kitty. She goes for checkups.’* Noa averted the spotlight by describing a typical veterinary visit for Apples, including that Apples dislikes getting shots. By describing Apples’ experience with and aversion to the vet, Noa skirted the potential social difficulty associated with describing their own experiences. At the same time, using Apples as a proxy, Noa communicated with clarity their feelings about healthcare.

Children enjoyed talking about animals during the interviews, and they described how animals helped them connect with others. Rain shared that she has a neighbor with a menagerie of animals– goats, chickens, bunnies—and she spends most afternoons there. Rain described knowing this neighbor well; she views them as a queer role model with whom she could relate to more deeply than many of her peers and family members. Children also shared that they confided in animals, sharing secrets that they did not feel comfortable sharing with others. *‘Sometimes I share secrets with Milly and Aster,’* June told us, referring to her pet rats. When we asked Mila for her final thoughts at the end of our interview, she responded, ‘*I think the doctor is fine, I just don’t like shots and all the stuff. Now show the picture of our dog, Mom!’* The topic of animals book-ended most of the interviews, facilitated rapport building, and was a source of judgement-free comfort that nearly all the children in the study benefited from in various ways.

#### Theme 3: ‘*I make those in my imagination*’: Windows into children’s lifeworlds

Can I show you how I draw things in my imagination?

I’m a ghost warrior. Someone who warriors the ghosts away.

I guess you do need a walkie talkie, so you could call.

I speak kitty, that’s how I can talk to Tooby and Baby Meowmeow.

I make them talk to other Legos. They usually talk about painting. They’re artists.

I drew Santa and Mrs. Claus because I love them.

I want to be Mrs. Claus when I grow up.

I’m going to draw a yellow wheeler.

I put a zombie inside. Look at my zombie.

I was a baby, so I probably thought it was a toy.

I make those in my imagination.

**Figure 4.**
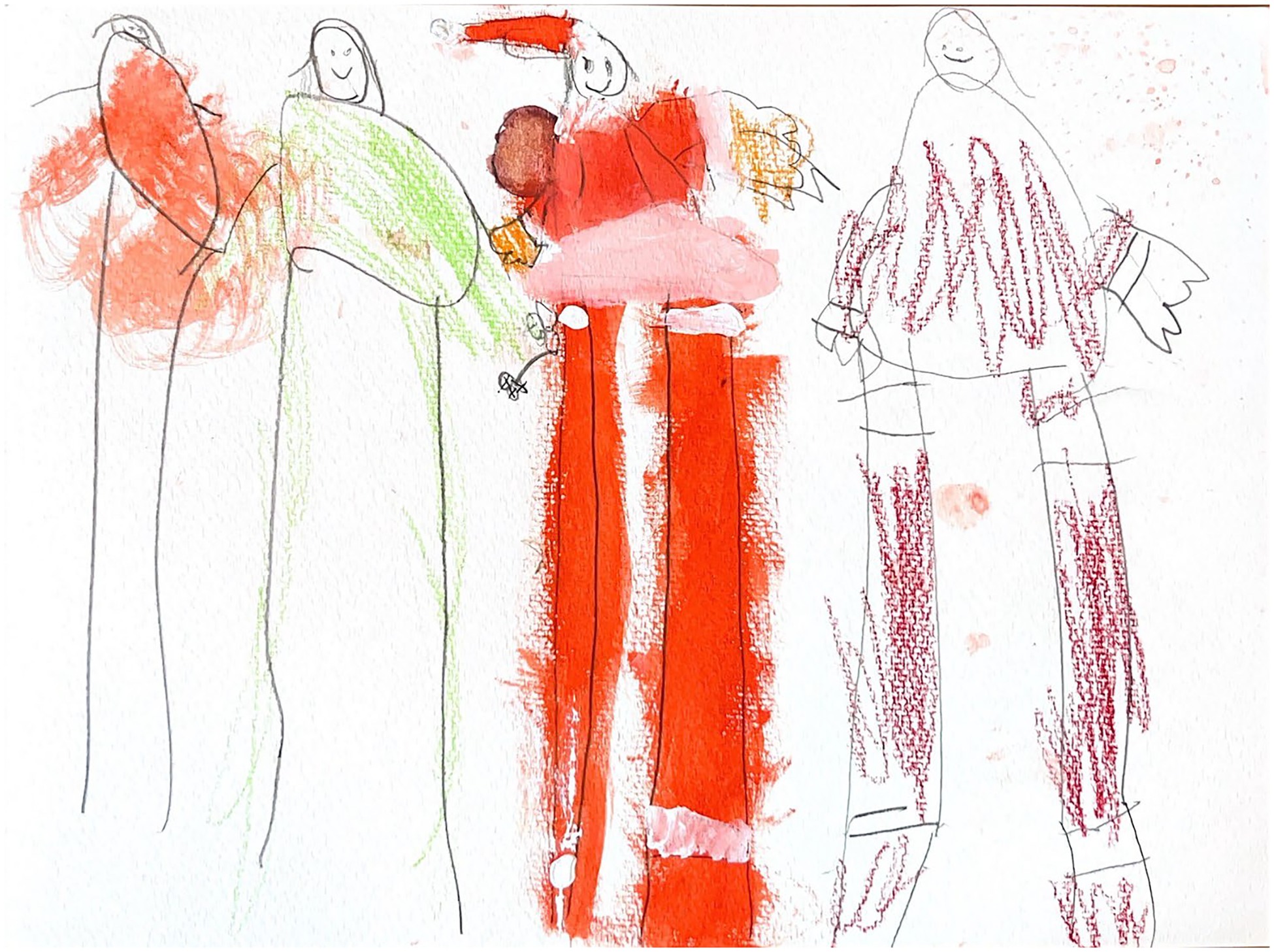
Skye (6) drew herself alongside her parent, Santa, and Mrs. Claus.

Children often vacillated between real and imagined experiences both within their drawings and in the conversations that followed. We generated a corresponding theme about their imaginations and how children invited us into their lifeworld^2^ through the draw-and-tell process. Their illustrations revealed the fluidity and creativity of their pre-reflective and lived experiences. In some cases, these were positions of resistance, for example when Alex drew Lego Dad and Baby in response to the first drawing prompt (i.e., draw the most important people in your life). *‘They’ve been in my house forever, and they’re my favorite Legos,’* she offered, choosing not to say anything about family members and friends in her life. Others, like Skye, used fictional characters to achieve certain connections. ‘*I drew Santa and Mrs. Claus because they’re real and important to me.’* Skye wants to be Mrs. Claus when she grows up, ‘*Because she’s a girl!’* Alex and Skye, as well as many of the others in the study, actively shaped their stories and redefined characters, relationships, and realities on their own terms. Their drawings reflected their emotions, relationships, and identities, and their narratives were often subversive as they actively negotiated truths and reconstructed meaning (Figure 4).

**Figure 5.**
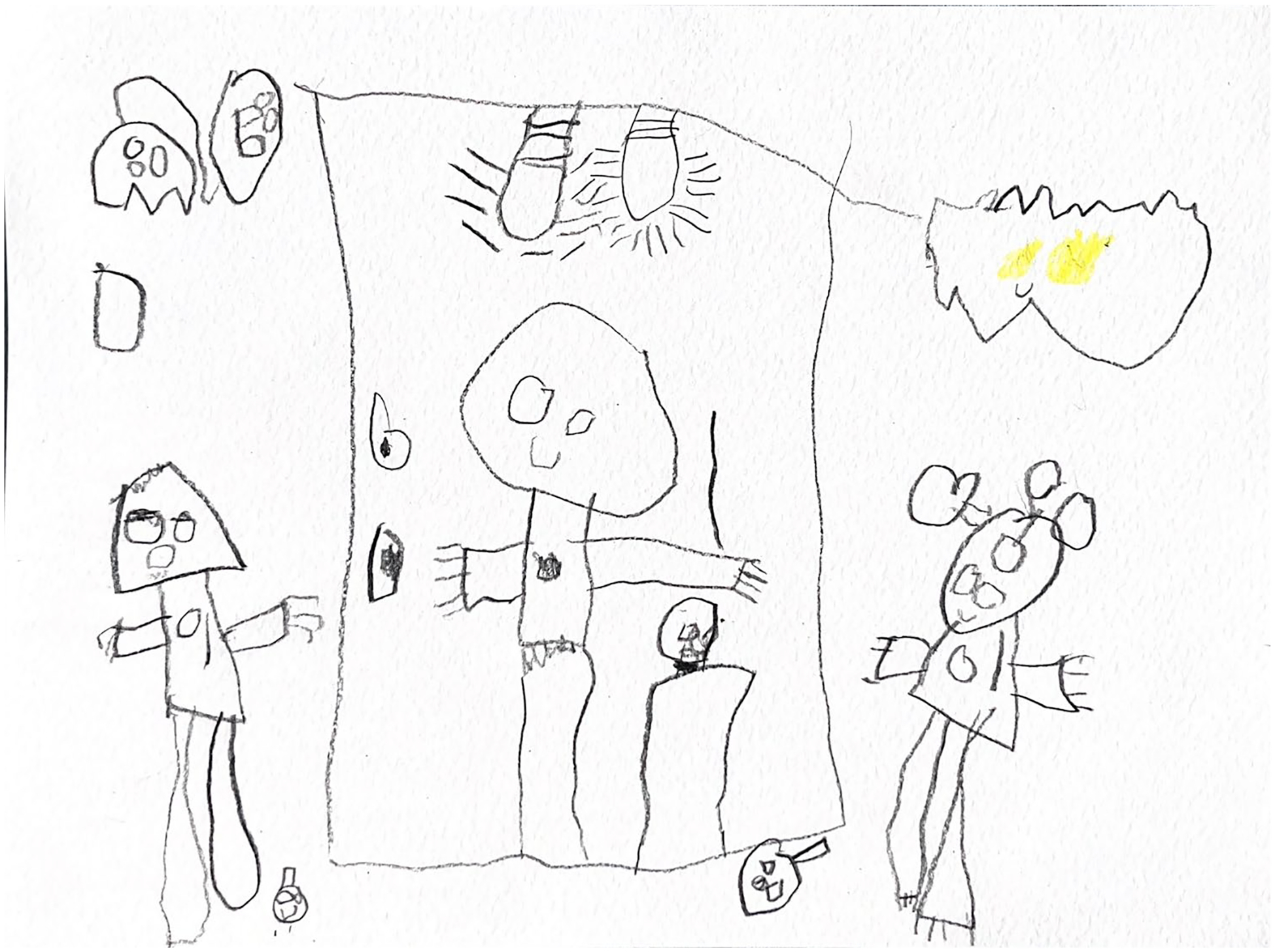
Sage (5) drew herself, her mom, and her dad as robots and zombies.

Sage described a character she drew as her mom, dressed up as a robot but then later in the interview, she decided that this character was instead her dad, dressed as a zombie. In this slippage, Sage discursively redefined the meaning of the signifier she had drawn, reconstructing what was her robot mom into her zombie dad and asserted her agency and imagination in the process. The line between the real and the imagined shifted in accordance with what Sage needed to communicate in the moment (Figure 5).

Other children also drew imaginary beings and settings instead of ‘real’ ones. Their responses to the interview questions often took the perspectives of the imaginary beings they drew or built, in some ways mirroring Noa’s earlier use of Apples the Cat. Two children refused to draw and proposed alternatives—one, Rory, wanted to build a Lego hospital and ambulance instead. We spent an hour building together– weaving questions into the imaginary play. Rory actively negotiated their agency during the session, choosing whether and how to respond.

Interviewer: Does that person have pronouns?

Rory: Yeah!

Interviewer: What are their pronouns?

Rory: Oh, uh, this is actually… A head. This is actually his hair. You’re finding all the pieces for this guy. This looks like a good nurse, doesn’t it?

At times, Rory was the one asking questions.

Rory: Beep beep beep. Oh! We need an ambulance door! Here’s one of those doors. Oh and here’s a breathing thing. Have you had one of those?

Interviewer: No, have you?

Rory: It helps you breathe when you can’t breathe.

Interviewer: What is it like to wear that? Is it scary?

Rory: No. It’s just like, it has like, it gives you air. It puts air into the lungs. Like the lungs are like, ‘I don’t want to work anymore’ and it pumps air in and it’s like, ‘Oh, I have to work now.’ Here’s some doors. I found doors!

**Figure 6.**
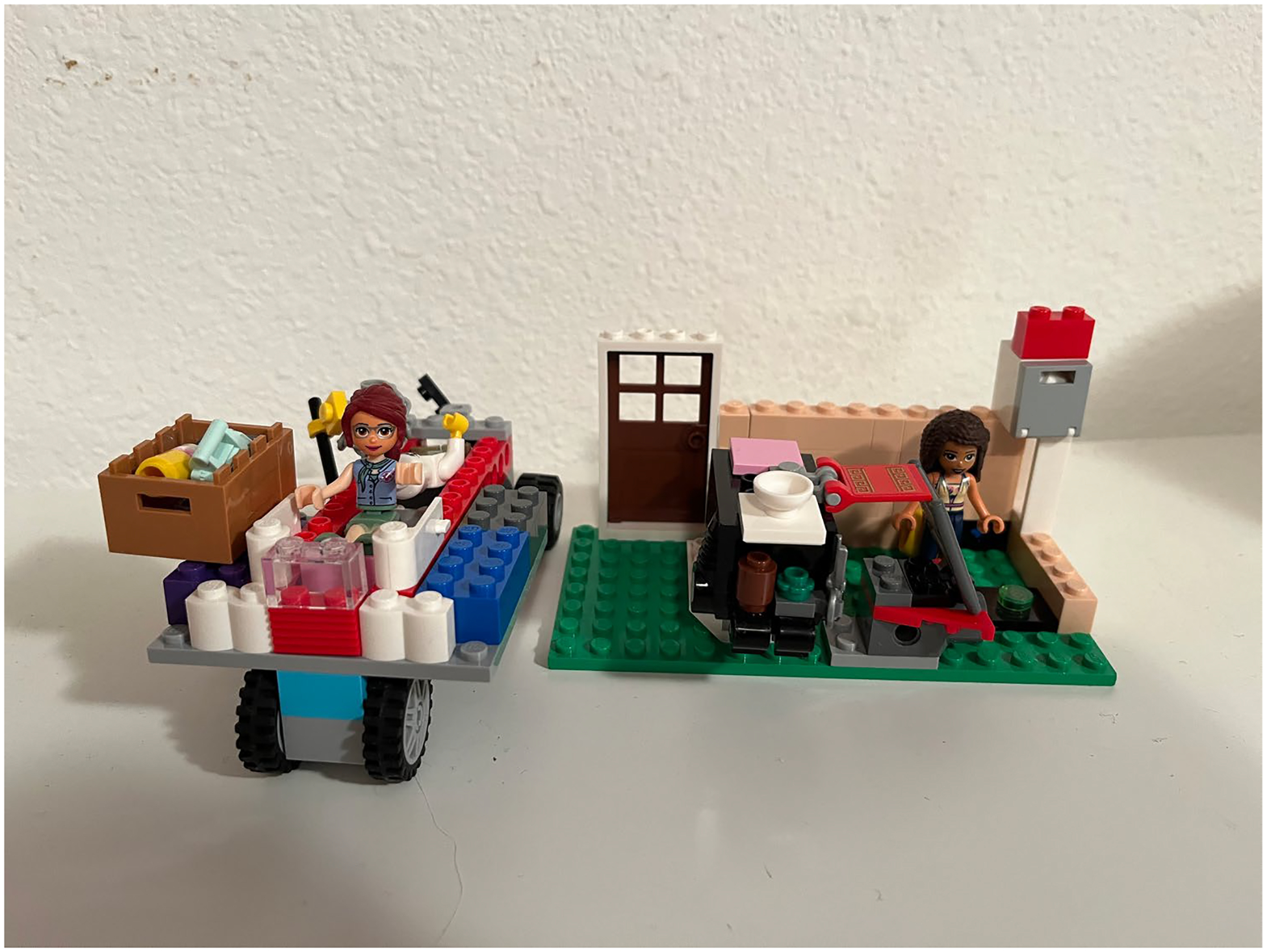
Rory (5) built a Lego hospital and ambulance.

In both excerpts, Rory created an imaginary environment in which to discuss both abstractions (e.g., ‘a good nurse’) and concrete experiences (e.g., the breathing machine) (Figure 6).

Another child, Rain, requested that we do the interview while walking in the woods. Rain described what she would have drawn: *‘A room with a chair in the middle. Usually. […] And it’s always super loud, like when you move the tiniest bit it’s just like, CRACCKKK!’* She stopped to study a mushroom growing on a nurse log before continuing, ‘*And more chairs. Usually uncomfortable. With the smallest amount of padding and thin arm rests and one big fancy chair in the middle for the patient.’* In conjuring a sterile and unpleasant clinical space, Rain offered us a stark contrast to the wooded environment where we stood. Her account reflects her embodied and sensorial memories, which, as van Manen (1997) suggests, are central to how children experience and give meaning to their lifeworlds.

#### Theme 4: ‘*I just want to tell you*’: Talking and not talking about gender

I don’t want to talk about this.

I just want to tell you.

I know who I am.

I get too uncomfortable around doctors.

It’s [like], Question. Question. Question. Question.

I would enter the room and they would be like, ‘how do you identify? What are your pronouns?

What is your chosen name?’

I want them to ask me because if they ask my parents then it will seem [like, like, like, um, like], that I don’t know how to answer.

I would want someone to ask me.

I’d want them to say something like, ‘what is your experience with gender?’

I wish they knew something about my body… that’s all I want to say.

I would say [like] what’s your gender or something before I tell them, [like].

I don’t want to share that myself, [like] just say that.

I talk about it with a friend sometimes, but not always, it’s hard to talk about.

I know that whenever a group is introducing each other

I usually say, ‘Hi I’m June, and my pronouns are they/them/theirs.’

I like my school cause it’s accepting of everyone.

This I-Poem illustrates participants’ shared tension about gender and disclosure. In a few cases, children felt comfortable talking openly about their gender, but most (*n* = 9; 75%) wanted to avoid the topic during the DTC interview and with other inquiring individuals in their daily lives. Although they did not want to discuss gender directly, they still responded to questions that spoke indirectly to gender. After children finished their first drawing we asked, *‘Does anyone here have pronouns?’* Children seemed to appreciate this question, which turned the spotlight away from themselves in a way that fostered openness and connection to others—everyone has pronouns. Most of the children answered easily, *‘[My friend] also uses they/them pronouns and they’re also nonbinary. [My other friend] and my teacher and mom all use she/her pronouns,’* Marlo shared. *‘[My sister] is she, Maman is she, and Monsieur A. is he,’* Kai shared.

**Figure 7.**
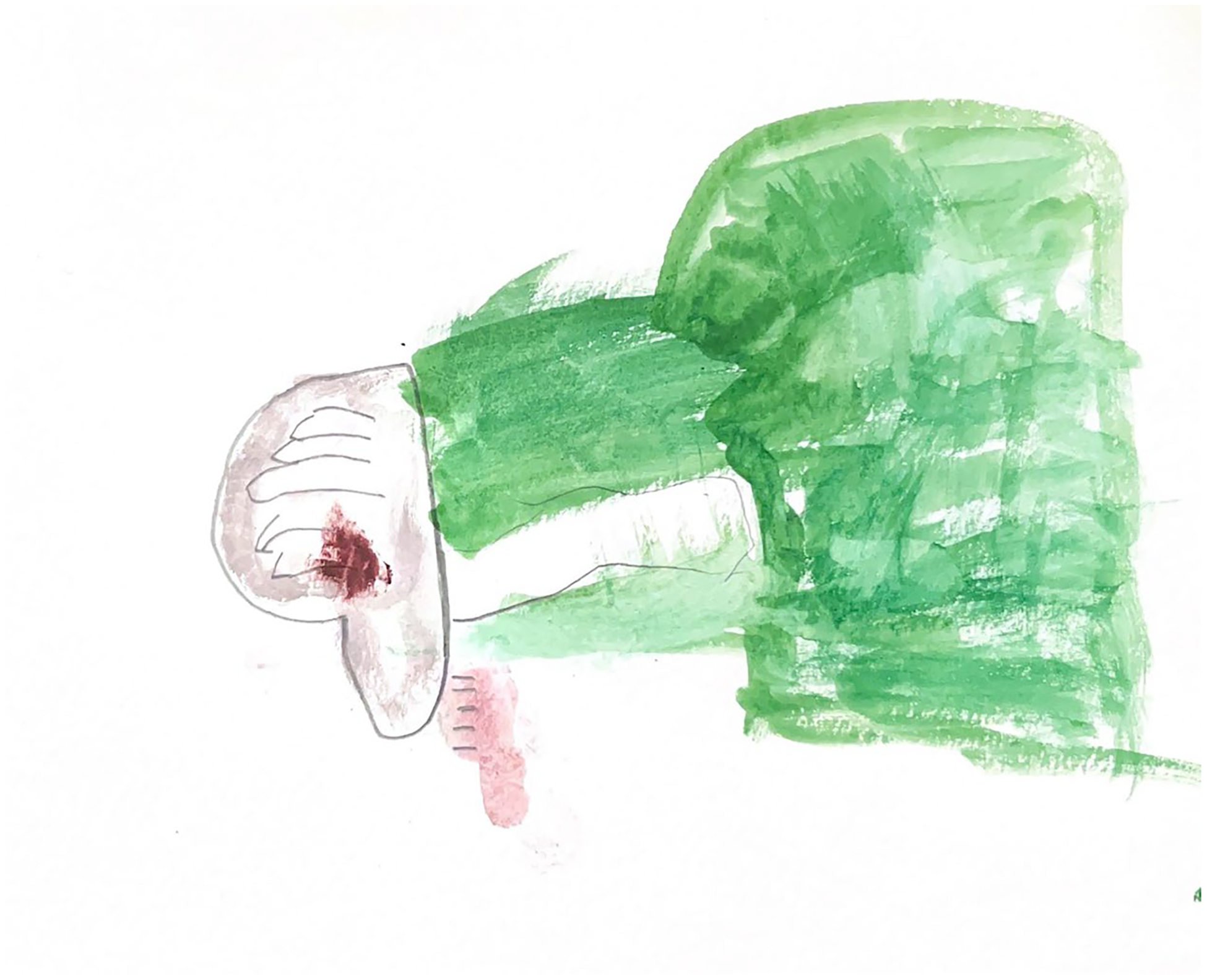
Charlie (8) drew two thumbs down, referring to his experience at the gender clinic.

Some asked their caregivers to respond to gender-related interview questions on their behalf, creating distance between themselves and their desired responses. *‘I don’t want to talk about this,’* Charlie mumbled while spinning in an office chair. ‘*Mama, you say it.’* His parent went on to say, *‘He’s basically just, he’s been set in his gender since two and a half, so he doesn’t really feel the need to talk about it.’* Other children changed the subject entirely, *‘Can I dump the dirty watercolor jar in the toilet*?’ Noa asked in response to a question about how talking about gender makes them feel (Figure 7).

Every child navigated their identity and subsequent expressions in two ways: (1) as an internal process and choice to reject essentialized and assigned labels in favor of something they felt deeply inside themselves; and (2) as a social process, in which they determined when, how, and with whom they expressed their gendered identities. Bringing adults along on their journey and helping them see who they are is both a sophisticated and challenging task; one that is particularly demanding for young children.

#### Theme 5: ‘*I just take deep breaths*’: Navigating clinical experiences

I usually just feel fine.

I went to the sleep study. I had to go to sleep… it was weird.

I woke up and everything was weird and not where I thought I’d be.

I was in a room that I did not imagine.

I liked the balloon blowing part.

I’m going to do the one that was most recent which was getting an x-ray of my toe.

I didn’t like it because I had to stand in an uncomfortable position.

I liked pretending that I’m the doctor before the doctor got there.

I got on the doctor’s stool.

I watched the computer and typed my name in as the password.

I got the thing around my wrist and told them why I was going there and there was a woman behind the screen here who was typing all the information into the computer to print into my bracelet.

I like when they do that thing that gets tighter, and tighter, and tighter.

I like when my mom is close by.

I liked him a lot.

I think the doctor is fine.

I just don’t like shots and all the stuff.

I don’t like getting shots and blood draws.

Sometimes when I do a shot,

I just take deep breaths and do it, but one time, one time,

I started crying and kicking and screaming and I hated it.

I looked at it directly.

I hate shots.

I never want to get one, but my friend who’s trans, she got her puberty blockers recently.

I just want them to be pills.

I don’t want to do injections!!

I don’t like when they have to take off your clothes.

I don’t like that cause you’re in public and the doctors have to see you naked.

That feels embarrassing.

**Figure 8.**
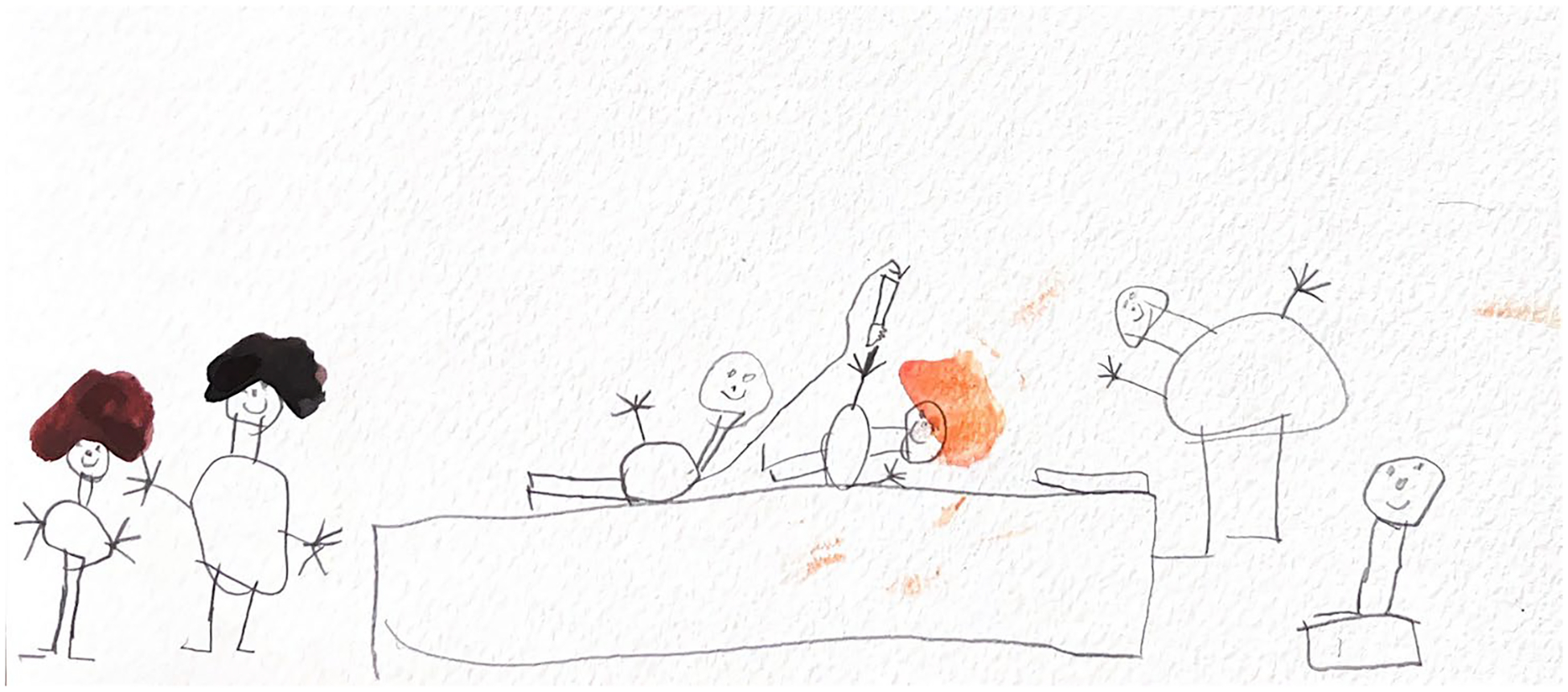
Kai (6) drew themselves lying on an operating table surrounded by providers. Their parents stand off to the side.

**Figure 9.**
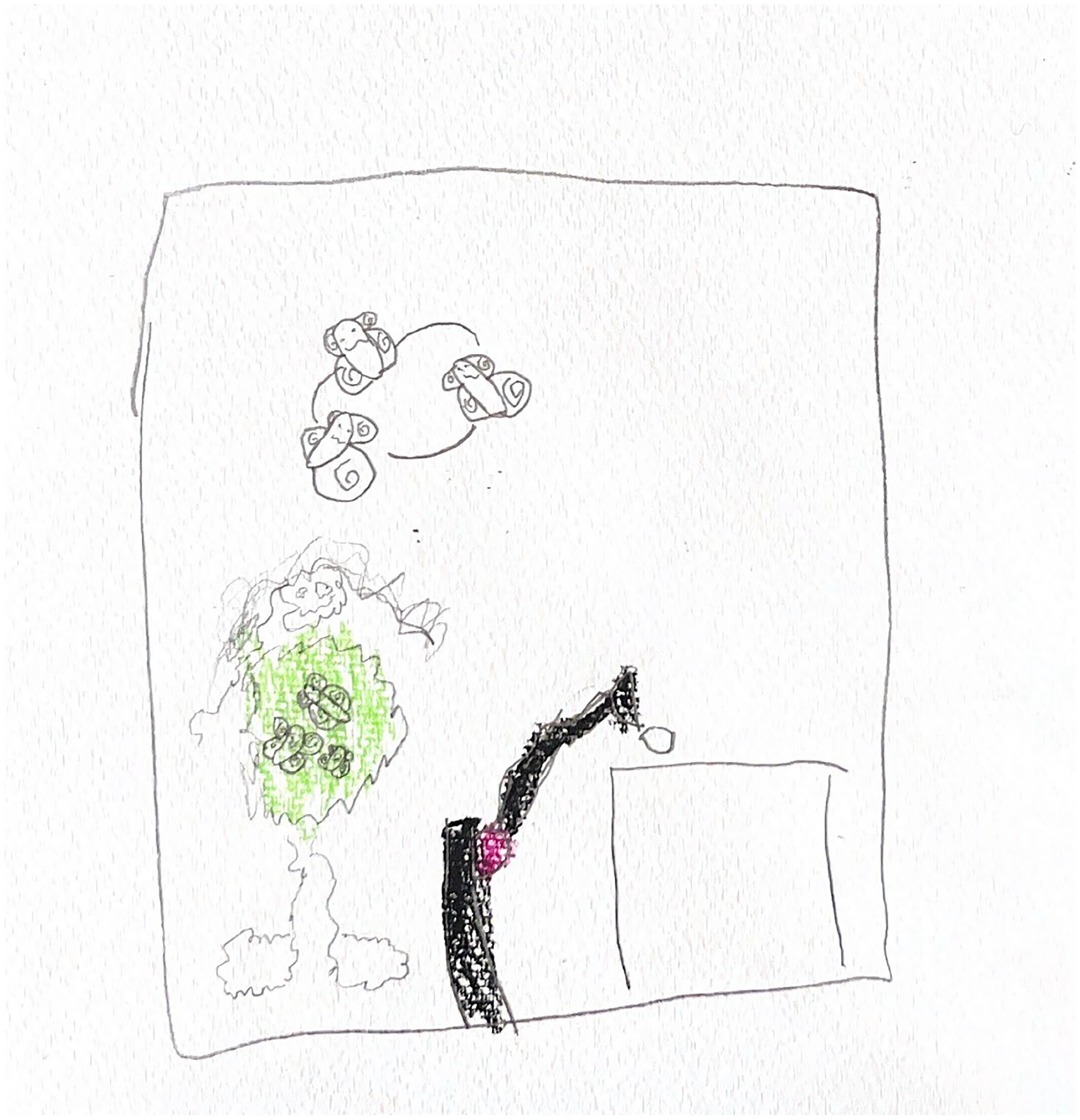
Alex (6) drew herself with butterflies in her stomach and flying over her head as she felt the anesthesia kick in prior to surgery.

This theme related how children experienced and subverted power in the clinical context. Most of the children (*n* = 9; 75%) depicted specialty visits (i.e., ER, surgeries, x-rays, etc.) rather than routine well-child visits. Some children illustrated their role within the clinical setting, showing their awareness of power dynamics (e.g., doctor on rolling chair vs. child stationary or a child on the exam table with the doctor towering over them). Others depicted external spaces (parking lots, hospital exteriors), possibly to distance themselves from clinical experiences, to assert their agency in choice of subject matter to depict, or simply because they found it more interesting (Figures 8 and 9).

Not dissimilar from Theme 4, participants described deliberate choices in how and what they communicated about themselves in the clinical setting. A few children preferred to have their primary caregivers communicate key information with their providers ahead of time (e.g., affirming pronoun and name), yet many preferred it when providers talked to them directly, validating their perspectives as autonomous individuals. June said they wanted to be treated like an expert by having their provider ask them questions directly, explaining, *‘*[…] *if they ask my parents then it will seem like […] that I don’t know how to answer.’*

**Figure 10.**
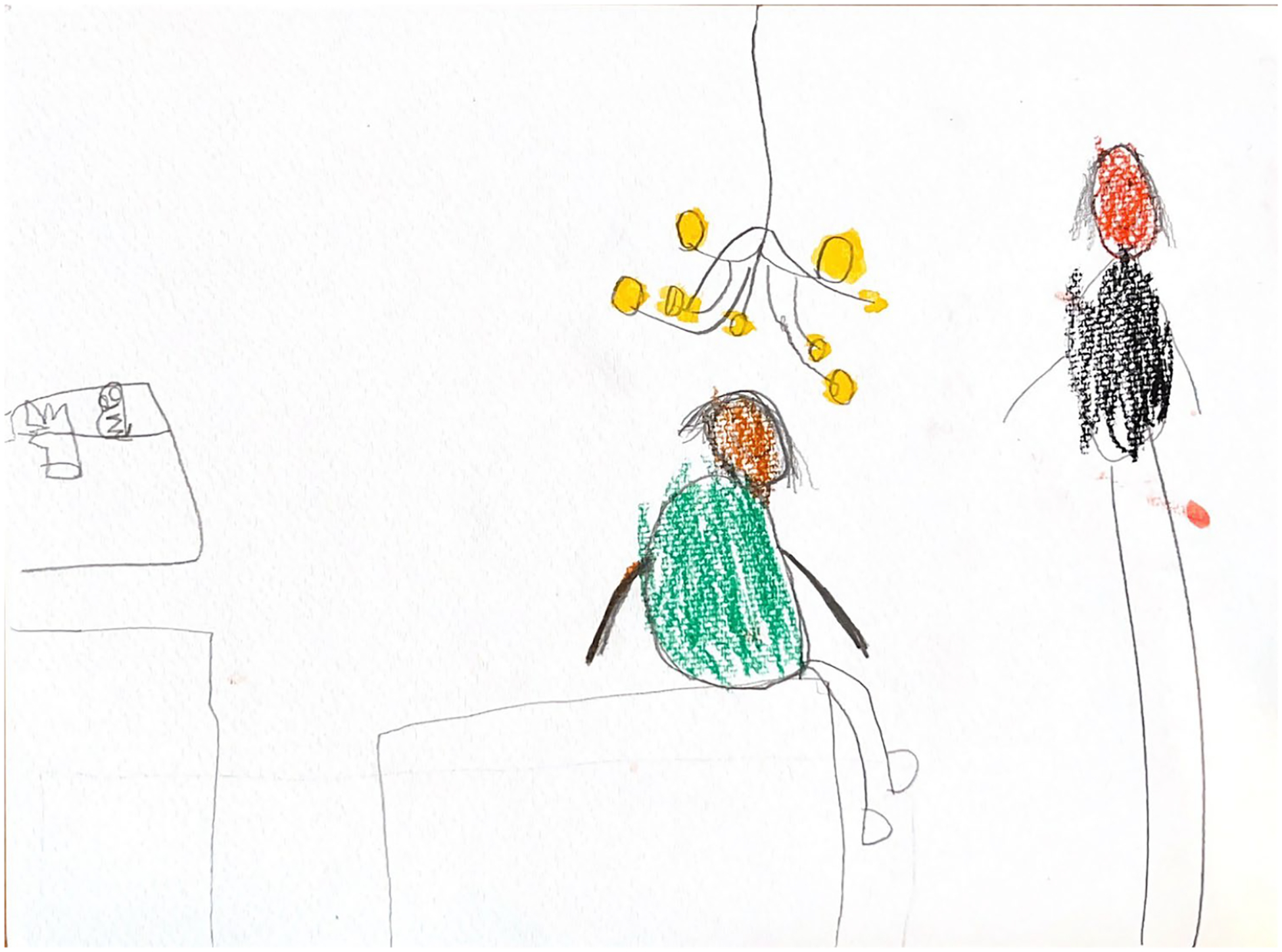
Skye (6) drew herself on an exam table with her pediatrician towering over her.

**Figure 11.**
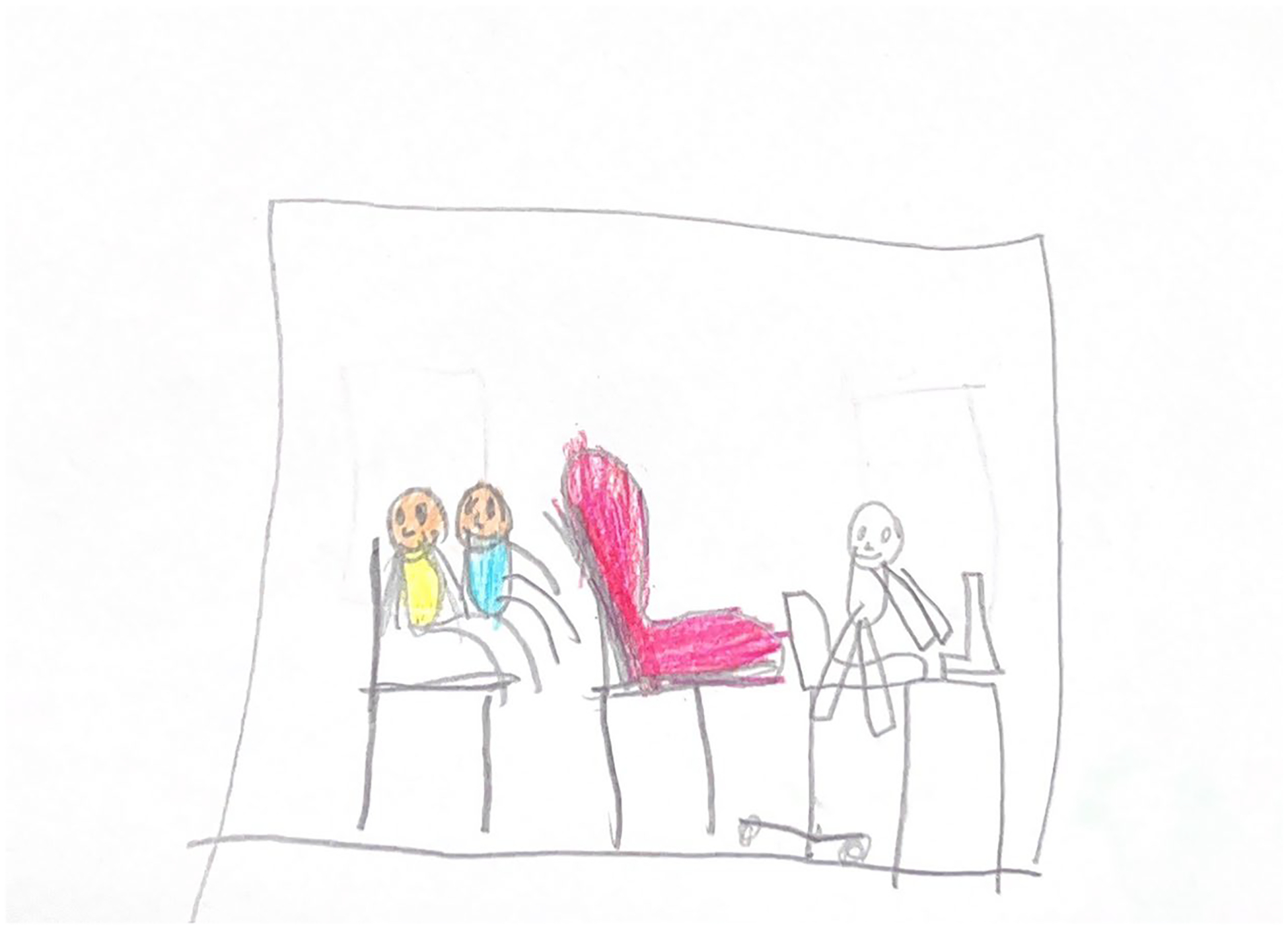
Mariel (6) drew themselves on their parent’s lap. There is a pink exam table in the middle of the room. On the other side of the room a doctor, seated in a rolling chair, types away on a computer.

Ten children (83.3%) expressed feeling a lack of power and agency in the presence of pediatric providers, four children (33%) shared experiences of being misgendered, seven children (58.3%) felt they were asked too many questions. In the survey, half the caregivers (*n* = 6; 50%) reported that gender came up during the most recent pediatric visit. Five (41.7%) said puberty came up in the most recent visit. Several children felt that they were not seen for who they are, or that they have been misunderstood their entire lives. ‘*The midwives made a mistake when I was a baby, they didn’t know I was a girl,’* Skye shared. Many children felt aware of their difference, and caregivers echoed this sentiment. One of the caregivers in the study shared, ‘*We feel like we’re probably [the pediatrician’s] first rodeo*.’ Others shared that they switched to a provider who was well-known in their community as being trans-affirming after several negative experiences with providers who did not demonstrate gender-affirming care. Although none of the children received gender-affirming medical care during this study, at least six (50%) of the children completed an initial intake visit at a gender clinic and three families (25%) received referrals to a gender clinic during their most recent visit (Figures 10 and 11).

While some participants chose to have their parents speak on their behalf, others liked being part of the conversation and being invited to share pronouns. They appreciated predictable routines, being near caregivers, and receiving treats after the visits. They enjoyed exploring the clinical tools, pretending to be the doctor while they waited, and multiple kids liked the blood pressure cuff, which Mila described as, *‘When they do that thing that gets tighter and tighter and tighter.’*

Children disliked lacking agency, being asked too many questions, having to undress, being misgendered, and getting shots. Sage, contrary to the others, did not like the blood pressure cuff at all, which she described as ‘*getting pumped up by air*.’

In the clinical context, although children wished to play an active role in their care, they also wanted their caregivers there to support and advocate for them, and caregivers had a crucial role in ensuring their children were affirmed in their gender within the clinical context. Several caregivers shared stories of providers misgendering their children and how they subsequently minimized the likelihood of repeat-events by introducing their child at the onset of every visit, finding more affirming providers, and calling the clinic ahead of time. At least two children in this study resisted attending subsequent pediatric visits because of past misgendering experiences.

## Discussion

A central aim of this study was to hear directly from gender-creative children. Our findings suggest that children know themselves and want to be supported as themselves, even as their sense of self shifts and deepens. Children want to be able to make their own decisions about how or whether they answer questions related to their gender. They did not want to be treated differently, singled out or probed more than their peers to explain things about themselves. Yet the children did want to be given the chance to share their chosen names and pronouns in circumstances where there is a risk for confusion. June, summarizing the sentiments across most of the interviews we conducted, stated: *‘Everyone should be able to access things for their body… it’s not fair that some people can access those things and others can’t.’* Gender-creative children deserve to be heard, protected, and celebrated—not erased. June and their gender-creative peers are not only affected by the political and medical systems, but they are also thinking critically and enacting their agency to effect change.

This study contributes to a growing body of research that directly engages transgender and gender-creative children’s perspectives and experiences. To date, systematic reviews have identified few qualitative studies centering prepubertal children’s voices (Horton, 2024). Our findings align with and extend existing work in several ways. Consistent with previous findings (Horton, 2022; Malpas, 2011; Olson et al., 2016; Parker & Davis-McCabe, 2021; Rafferty, 2018)), our participants emphasized the critical importance of family support and acceptance. However, our study is distinct in showing how young children experience and articulate this support through embodied and relational metaphors, for example when Mila described the sun in her drawing as being symbolic of her ‘warm and loving’ family. Children lean on several important people for support, care, and guidance; namely primary caregivers, close friends, siblings, and teachers. In the presence of these loved ones, children talk more freely about gender, their bodies, and their health, topics that they did not enjoy discussing more broadly.

The prominence of animals as sources of social support for gender-creative children represents a novel contribution to the literature. Previous research has documented the importance of human social networks for gender-creative children (Ehrensaft, 2016; Rafferty, 2018)), as well as the positive ‘pet effect’ on transgender adults (Grey et al., 2024). The children in our study spontaneously and enthusiastically described animals as trusted confidants, sources of comfort, and facilitators of social connection, suggesting an opportunity for researchers and clinicians to consider animals as part of gender-creative children’s support systems.

Poststructuralist, queer, and trans theories provided a valuable lens for interpreting how participants resisted normative pressures and authored new expressions of self. Rather than conforming to imposed categories, the children reimagined and queered^3^ their identities on their own terms. The sensorial and pre-reflective ways of knowing evident throughout our findings demonstrate how children’s lifeworlds are constituted through imagination, play, and fluidity between real and imagined experiences (van Manen, 1997). The children actively negotiated meaning through their drawings and narratives, exercising agency in how they represented their identities and relationships. Poststructuralist theories of fluidity offered a valuable lens for understanding the dynamic ways in which children come to know and express themselves (Weedon, 1987). The children’s expressions of self were not rooted in fixed or essentialist notions of identity, but emerged ongoing processes of negotiation, meaning-making, and self-articulation (Weedon, 1987; Zaman & Anderson-Nathe, 2021). Their self-expressions were not static but continually shaped and reshaped through their interactions with social context, power, and discourse (Weedon, 1987).

Children’s ambivalence about discussing gender both echo and complicate existing research (Olson et al., 2019; Riley et al., 2011). Studies with transgender youth have documented how disclosure is a complex and ongoing process shaped by context and relationship (Medico et al., 2020). Our participants demonstrated a precocious ability to navigate when, how, and with whom they shared their gender identities. Their preference for indirect questions (e.g., ‘Does anyone here have pronouns?’) that acknowledge the universality of gender rather than spotlight their difference offers important methodological and clinical insights. This finding challenges deficit-based framings that pathologize gender-creative children’s reluctance to discuss gender (Horton, 2023), instead highlighting their strategic agency in managing social dynamics and protecting themselves from potential harm.

The clinical experiences children described suggest opportunities to improve pediatric care practices. Participants described feeling disempowered, over-questioned, and sometimes misgendered in clinical settings. Five participants in the study identified as non-binary, and four as trans female. These identity groups are noteworthy in the clinical context, as they are the most frequently misunderstood and misrepresented populations in healthcare settings. Healthcare providers often have limited training or familiarity with the specific needs, language, and lived experiences and associated with these identities (Berrian et al., 2025), potentially compounding existing feelings of invisibility or otherness that non-binary and trans female youth already disproportionately encounter in broader social contexts compared to other LGBTQ+ identities (Bower-Brown, Zadeh, & Jadva, 2023). The discomfort participants expressed in the clinical encounters may be especially acute for those whose identities are least likely to be met with familiarity or affirmation.

The predominance of specialty visits rather than routine well-child visits in the children’s drawings may reflect their interest in more acute medical events, and perhaps a feeling of being attended to more directly (e.g., participating in a sleep study and getting to explore all the associated medical equipment or getting a cast for an injured ankle) over preventative care visits in which they may feel less directly engaged in the content of the visit (e.g., witnessing their provider and caregiver discuss their development as though a fly on the wall) while simultaneously feeling more interrogated (e.g., being asked to describe their experience with gender while a provider takes notes). It is notable that children in our study identified teachers with whom they felt connected with, but did not identify healthcare providers. Despite increased clinical attention to gender-creative children in clinical practice, current approaches to hearing from children may be improved through the adoption of tools and strategies that are more child-focused and led, such as the use of drawings to help stimulate or expand upon conversations (Driessnack, 2005).

This is one of the first studies that directly engages younger gender-creative children, so there are ample opportunities to expand on this work. There is a clear opportunity for providers to build trust with children in clinical settings and support families in determining how to support children’s sense of autonomy and agency in the process (Ehrensaft, 2016; Ehrensaft et al., 2018). Children’s sense of safety, trust, and belonging within the medical context is greatly impacted by how they are affirmed (Durwood et al., 2021; Ehrensaft et al., 2018). Rather than focusing narrowly on gender identity development or medical transition pathways, our results suggest that children benefit from holistic support that acknowledges their full lifeworlds, including their relationships with family, friends, teachers, and even animals; their imaginative lives; and their need for agency and autonomy in clinical encounters. The emphasis children placed on being asked questions directly, being seen as experts on their own experiences, and having caregivers present to support (rather than speak for) them offers concrete guidance for developing more affirming clinical practices and reaffirms the importance of child voice in healthcare and research (Horton, 2024; Tyler et al., 2025). The children offered examples of questions providers could pose about pronouns, what would be helpful as they prepare to visit a clinic for the first time, and how they would like the clinic experience to feel (Table 1).

Our study’s participatory, arts-based methodology allowed children to communicate on their own terms, shifting between literal and metaphorical expression, using proxies like pets to discuss sensitive topics, and actively shaping the interview process through their choices. This approach yielded insights that might have been inaccessible through more traditional interview methods, particularly given many participants’ reluctance to discuss gender directly. The drawings gave us a starting point, something to talk about and return to when children felt stuck or did not know what to say. They helped redirect the focus away from the child and allowed space to formulate ideas before saying them out loud (Driessnack, 2005). The fact that children could choose to draw whatever they wanted, or even choose not to draw at all, helped them feel a sense of control. The DTC is a powerful method for engaging with younger children because it is designed to meet children in their current stage of development (Driessnack, 2006; Pope et al., 2018). In some cases, the drawings included imaginative elements, offering a lens into children’s lifeworlds as framed by phenomenologists Husserl (1970), Gadamer, and van Manen (1997). The lifeworld describes those pre-reflective experiences and contexts that shape how people perceive and engage with the world (Schutz & Luckmann, 1973). From a post-structuralist perspective, the depicted lifeworlds constitute discursive formations through which to interpret the children’s experiences (Belsey, 2022; Husserl, 1970; Van Manen, 1997).

I-poems captured the general sentiments spontaneously expressed across the interviews. They act as both an analytic method that aids in the sense-making of a set of texturally unique interviews, and as a way in which to present the data (Nicolaou & Eloff, 2024). Introducing findings as I-poems centers children’s voices and contradictory, layered, and complex insights (Nicolaou & Eloff, 2024).

### Limitations

We made choices to account for children’s vulnerability and safety, and these choices resulted in a series of limitations. Most of the participants came from affluent, white families. All participants lived in politically liberal enclaves, and this may have influenced the support they received from family members, teachers, friends, and pediatric providers. Most participants (*n* = 9/12) identified as non-binary or trans female; identities that are poorly understood and discriminated against by society to a greater extent than identities such as trans male. Given these limitations, the findings are limited in their generalizability. Our personal biases could also be interpreted as limitations—we approached the research *via* lived experiences that influenced the questions asked and the methodological approach. As insiders, we could build trust with children and caregivers quickly and we believe that the results are richer because of this. However, our shared experiences may also have influenced what was and was not said due to an assumed mutual understanding.

## Conclusion

This study highlights the complex ways in which gender-creative children engage with and make sense of their social worlds. Through their drawings and narratives, participants contested normative gender binaries and articulated embodied experiences that are both imaginative and deeply reflective. The Draw-and-Tell Conversations method offered a participatory framework through which children could assert their autonomy and co-construct meaning. Their accounts further revealed an awareness of interpersonal and institutional dynamics, particularly within pediatric and social contexts. These findings underscore the importance of treating gender-creative children as knowledgeable social actors. For healthcare providers and caregivers alike, this necessitates an ongoing commitment to listening, affirming, and adapting in response to children’s evolving identities and needs.

## Figures and Tables

**Table 1.** Gender-creative children’s recommendations for providers.

Recommendation	Description
Introduce social stories	Providing children with a social story that outlines what they can expect to do during their visit can help reduce anticipatory anxiety and help them feel more comfortable.
Share pronouns	Children want providers to share their own pronouns and ask, “Does anyone here have pronouns?” or “What’s your experience with gender?” By inviting everyone in the room to share pronouns if they felt comfortable takes the pressure off the individual child.
Space to be me	Children want space to be themselves. They want to be talked to directly and they want to weigh in on decisions. They want providers to follow their lead and offer scaffolding to broach difficult topics.
Opportunities to play	Children want more opportunities to play, and this will lead to increased trust as they come to see providers not only as clinical workers who do things *to* them (e.g., poke, prod, measure, test) but also as fun adults who do things *with* them (e.g., build puzzles, play games, draw pictures).
More choices	Children want more choices. Choices in where to sit, what to do during the visit (e.g., draw; build with Legos, Magna tiles, blocks; sculpt out of modeling clay; etc.), what part of the visit to do first (e.g., check ears or heart first).
Fewer questions	Children want to be asked fewer questions. Instead, providers may rely more on chart review and possibly separate conversations with caregivers that children consent to

## Footnotes

1. We use the term transgender to describe individuals whose gender identities “differ from the gender socially attributed to the sex assigned to them at birth” (Coleman, 2022, p. 55). We use the term gender-creative to refer to children who creatively play with their gender identity in ways that deviate from social norms and from their gender assigned at birth (Ehrensaft, 2016).

2. We use “lifeworld” to refer to the pre-reflective state that is experienced in daily life, as described by Van Manen (1997), Gadamer (1976), and Husserl (1970).

3. We use “queered” as a verb referring to the practice of rejecting gender essentialism and deliberately unsettling or queering the taken-for-granted.

## References

[R1] Allen BJ, Coles MS, & Montano GT (2019). A call to improve guidelines for transgender health and well-being: Promoting youth-centered and gender-inclusive care. The Journal of Adolescent Health, 65(4), 443–445. 10.1016/j.jadohealth.2019.07.020 31542110

[R2] Barfield PA, & Driessnack M (2018). Children with ADHD draw-and-tell about what makes their life really good. Journal for Specialists in Pediatric Nursing, 23(2), e12210. 10.1111/jspn.12210 29489068

[R3] Belsey C (2022). Post-structuralism: A Very Short Introduction (2nd ed.). Oxford University Press.

[R4] Berrian K, Exsted MD, Lampe NM, Pease SL, & Akré E-RL (2025). Barriers to quality healthcare among transgender and gender nonconforming adults. Health Services Research, 60(1), e14362. 10.1111/1475-6773.14362 38988141 PMC11782051

[R5] Bower-Brown S, Zadeh S, & Jadva V (2023). Binary-trans, non-binary and gender-questioning adolescents’ experiences in UK schools. Journal of LGBT Youth, 20(1), 74–92. 10.1080/19361653.2021.1873215 41141326 PMC7618297

[R6] Braun V, & Clarke V (2021). Can I use TA? Should I use TA? Should I *not* use TA? Comparing reflexive thematic analysis and other pattern-based qualitative analytic approaches. Counselling and Psychotherapy Research, 21(1), 37–47. 10.1002/capr.12360

[R7] Bravo T (2025, February). ‘There is no LGB without the T’: S.F. join fight against Trump to stop trans erasure. San Francisco Chronicle. Retrieved May 26, 2025. https://www.sfchronicle.com/entertainment/article/lgbtq-erasure-trump-sf-20168220.php

[R8] Butler J (1990). Gender trouble: Feminism and the subversion of identity. Routledge.

[R9] Butler J (1993). Bodies that matter: On the discursive limits of ‘sex’. Routledge.

[R10] Butler J (2024). Who’s afraid of gender? Penguin Press.

[R11] Chang CJ, Kellerman J, Feinstein BA, Selby EA, & Goldbach JT (2022). Greater minority stress is associated with lower intentions to disclose suicidal thoughts among LGBTQ+ youth. Archives of Suicide Research, 26(2), 626–640. 10.1080/13811118.2020.1818656 32970971

[R12] Coleman E, Radix AE, Bouman WP, Brown GR, de Vries ALC, Deutsch MB, Ettner R, Fraser L, Goodman M, Green J, Hancock AB, Johnson TW, Karasic DH, Knudson GA, Leibowitz SF, Meyer-Bahlburg HFL, Monstrey SJ, Motmans J, Nahata L, Adelson SL, Alizai NK, Bakker A, Baldwin A, Beek TF, van der Miesen AIR, Bockting WO, Bouman M-B, Bradley SJ, Dhejne C, Fisher AD, Garcia MM, Hembree WC, Johnson KC, Klink DT, Kreukels BPC, Marshall E, Meyer WJ 3rd, Nieder TO, Reisner SL, Safer JD, Tangpricha V, T’Sjoen GG, Winter S, & Arcelus J (2022). Standards of care for the health of transgender and gender diverse people, version 8. International Journal of Transgender Health, 23(sup1), S1–S259. 10.1080/26895269.2022.2100644 36238954 PMC9553112

[R13] Coyne I, Mallon D, & Chubb E (2021). Research with young children: Exploring the methodological advantages and challenges of using hand puppets and draw and tell. Children & Society, 35(5), 813–830. 10.1111/chso.12452

[R14] Davies C, Waters D, & Fraser J (2024). Children’s and young people’s experiences of expressing their views and having them heard in health care: A deductive qualitative content analysis. Journal of Clinical Nursing, 33(4), 1506–1519. 10.1111/jocn.16952 38041392

[R15] Denton JM, & Cain LK (2023). Creating queer epistemologies and embodied knowledge through narrative and arts-based research. Departures in Critical Qualitative Research, 12(4), 133–157. 10.1525/dcqr.2023.12.4.133

[R16] de Vries AL, McGuire JK, Steensma TD, Wagenaar EC, Doreleijers TA, & Cohen-Kettenis PT (2014). Young adult psychological outcome after puberty suppression and gender reassignment. Pediatrics, 134(4), 696–704. 10.1542/peds.2013-2958 25201798

[R17] di Giacomo E, Krausz M, Colmegna F, Aspesi F, & Clerici M (2018). Estimating the risk of attempted suicide among sexual minority youths: A systematic review and meta-analysis. JAMA Pediatrics, 172(12), 1145–1152. 10.1001/jamapediatrics.2018.2731 30304350 PMC6583682

[R18] Driessnack M (2005). Children’s drawings as facilitators of communication: A meta-analysis. Journal of Pediatric Nursing, 20(6), 415–423. 10.1016/j.pedn.2005.03.011 16298282

[R19] Driessnack M (2006). Draw-and-Tell conversations with children about fear. Qualitative Health Research, 16(10), 1414–1435. 10.1177/1049732306294127 17079802

[R20] Driessnack M, & Gallo AM (2013). Children ‘draw-and-tell’ their knowledge of genetics. Pediatric Nursing, 39(4), 173–180. 24027951

[R21] Durwood L, Eisner L, Fladeboe K, Ji C, Barney S, McLaughlin KA, & Olson KR (2021). Social support and internalizing psychopathology in transgender youth. Journal of Youth and Adolescence, 50(5), 841–854. 10.1007/s10964-020-01391-y 33575917 PMC8272454

[R22] Durwood L, McLaughlin KA, & Olson KR (2017). Mental Health and Self-Worth in Socially Transitioned Transgender Youth. Journal of the American Academy of Child and Adolescent Psychiatry, 56(2), 116–123.e2. 10.1016/j.jaac.2016.10.016 28117057 PMC5302003

[R23] Ehrensaft D (2016). The Gender-creative child: Pathways for nurturing and supporting children who live outside gender boxes. The Experiment.

[R24] Ehrensaft D, Giammattei SV, Storck K, Tishelman AC, & St. Amand C (2018). Prepubertal social gender transitions: What we know; what we can learn—A view from a gender affirmative lens. International Journal of Transgenderism, 19(2), 251–268. 10.1080/15532739.2017.1414649

[R25] Fussell JJ (2011). The Pediatrician’s Role in Family Support and Family Support Programs. Pediatrics, 128(6), e1680–e1684. 10.1542/peds.2011-2664 22123873

[R26] Gadamer HG (1976). Philosophical hermeneutics. University of California Press.

[R27] Gill-Peterson J (2018). Histories of the Transgender Child. University of Minnesota Press.

[R28] Grey GE, Treharne GJ, Riggs DW, Fuller KA, Taylor N, & Fraser H (2024). The ‘pet effect’ and trans people: Associations between living with animal companions and wellbeing, social support, and trans-related marginalization in three international studies. International Journal of Transgender Health, 25(4), 694–703. 10.1080/26895269.2023.2234383 39465089 PMC11500547

[R29] Hesse-Biber S (2017). The practice of qualitative research (Third). SAGE Publications, Inc.

[R30] Horton C (2022). “I Was Losing That Sense of Her Being Happy”—Trans Children and Delaying Social Transition. LGBTQ+ Family: An Interdisciplinary Journal, 18(2), 187–203. 10.1080/27703371.2022.2076002

[R31] Horton C (2023). Depathologising diversity: Trans children and families’ experiences of pathologisation in the UK. Children & Society, 37(3), 753–770. 10.1111/chso.12625

[R32] Horton C (2024). The importance of child voice in trans health research: A critical review of research on social transition and well-being in trans children. International Journal of Transgender Health, 25(3), 389–406. 10.1080/26895269.2023.2295381

[R33] Husserl E (1970). The crisis of European sciences and transcendental phenomenology: An introduction to phenomenological philosophy (D. Carr, Trans.). Northwestern University Press. (Original work published 1936).

[R34] Jagose A (1996). Queer theory: An introduction. New York University Press.

[R35] Katz-Wise SL, Gordon AR, Sharp KJ, Johnson NP, & Hart LM (2022). Developing Parenting Guidelines to Support Transgender and Gender Diverse Children’s Well-being. Pediatrics, 150(3), e2021055347. 10.1542/peds.2021-055347 36045300

[R36] Kcomt L, Gorey KM, Barrett BJ, & McCabe SE (2020). Healthcare avoidance due to anticipated discrimination among transgender people: A call to create trans-affirmative environments. SSM – Population Health, 11, 100608. 10.1016/j.ssmph.2020.100608 32529022 PMC7276492

[R37] Kim JS (2023). Children’s experiences of intravenous injection using the draw, write, and tell method: A mixed-methods study. Journal of Pediatric Nursing, 71, 14–22. 10.1016/j.pedn.2023.03.005 36958135

[R38] Kitts RL (2010). Barriers to optimal care between physicians and lesbian, gay, bisexual, transgender, and questioning adolescent patients. Journal of Homosexuality, 57(6), 730–747. 10.1080/00918369.2010.485872 20582799

[R39] Laserna CM, Seih Y-T, & Pennebaker JW (2014). Um… Who like says you know: Filler word use as a function of age, gender, and personality. Journal of Language and Social Psychology, 33(3), 328–338. 10.1177/0261927X14526993

[R40] Lena SM, Wiebe T, Ingram S, & Jabbour M (2002). Pediatricians’ knowledge, perceptions, and attitudes towards providing health care for lesbian, gay, and bisexual adolescents. Annals (Royal College of Physicians and Surgeons of Canada), 35(7), 406–410. 12814100

[R41] Lenne E, Sun CJ, & Klawetter S (2023). An examination of power in a triadic model of parent–child–pediatrician relationships related to early childhood gender development. Journal of Family Theory & Review, 15(4), 662–676. 10.1111/jftr.12527 38351982 PMC10861221

[R42] Lesser JG (1999). When your son becomes your daughter: A mother’s adjustment to a transgender child. Families in Society, 80(2), 182–189. 10.1606/1044-3894.660

[R43] Lillemoe J, Holmstrom SE, & Sojar SH (2023). Emergency care considerations for transgender and gender diverse youth: A review to improve health trajectories. Current Opinion in Pediatrics, 35 (3), 331–336. 10.1097/MOP.0000000000001239 36876657

[R44] Linder LA, Bratton H, Nguyen A, Parker K, & Wawrzynski SE (2018). Symptoms and self-management strategies identified by children with cancer using draw-and-tell interviews. Oncology Nursing Forum, 45(3), 290–300. 10.1188/18.ONF.290-300 29683122 PMC6343495

[R45] Love H (2014). Queer. TSQ: Transgender Studies Quarterly, 1(1–2), 172–176. 10.1215/23289252-2399938

[R46] Malpas J (2011). Between pink and blue: A multi-dimensional family approach to gender nonconforming children and their families. Family Process, 50(4), 453–470. 10.1111/j.1545-5300.2011.01371.x 22145719

[R47] Marcus J (2025, January). Trump ‘gender ideology’ executive orders seek to deny existence of trans people and end DEI. Independent UK Edition. Retrieved May 26, 2025. https://www.independent.co.uk/news/world/americas/us-politics/trump-executive-order-gender-lgbtq-b2683184.html

[R48] Medico D, Pullen Sansfaçon A, Zufferey A, Galantino G, Bosom M, & Suerich-Gulick F (2020). Pathways to gender affirmation in trans youth: A qualitative and participative study with youth and their parents. Clinical Child Psychology and Psychiatry, 25(4), 1002–1014. 10.1177/1359104520938427 32638624

[R49] Nathan S, Hodgins M, Wirth J, Ramirez J, Walker N, & Cullen P (2023). The use of arts-based methodologies and methods with young people with complex psychosocial needs: A systematic narrative review. Health Expectations : An International Journal of Public Participation in Health Care and Health Policy, 26(2), 795–805. 10.1111/hex.13705 36628644 PMC10010092

[R50] McNamara M, Baker K, Connelly K, Janssen A, Olson-Kennedy J, Pang KC, Scheim A, Turban J, & Alstott A (2024). An Evidence-Based Critique of “The Cass Review” on Gender-affirming Care for Adolescent Gender Dysphoria. Yale Law School. https://law.yale.edu/sites/default/files/documents/integrity-project_cass-response.pdf

[R51] Nicolaou AM, & Eloff I (2024). The utility of I-poems to explore subjective well-being of children and adolescents with ADHD. International Journal of Qualitative Methods, 23(3), 48. 10.1177/16094069241241148

[R52] Olezeski CL, Pariseau EM, Bamatter WP, & Tishelman AC (2020). Assessing gender in young children: Constructs and considerations. Psychology of Sexual Orientation and Gender Diversity, 7(3), 293–303. 10.1037/sgd0000381

[R53] Olson KR, Blotner C, Alonso D, Lewis K, Edwards D, & Durwood L (2019). Family discussions of early childhood social transitions. Clinical Practice in Pediatric Psychology, 7(3), 229–240. 10.1037/cpp0000289 32864282 PMC7453930

[R54] Olson KR, Durwood L, DeMeules M, & McLaughlin KA (2016). Mental Health of Transgender Children Who Are Supported in Their Identities. Pediatrics, 137(3), e20153223. 10.1542/peds.2015-3223 26921285 PMC4771131

[R55] Olson KR, Key AC, & Eaton NR (2015). Gender Cognition in Transgender Children. Psychological Science, 26(4), 467–474. 10.1177/0956797614568156 25749700

[R56] Parker E, & Davis-McCabe C (2021). The sibling experience: Growing up with a trans sibling. Australian Journal of Psychology, 73(2), 188–199. 10.1080/00049530.2021.1882269

[R57] Pope N, Tallon M, Leslie G, & Wilson S (2018). Ask me: Children’s experiences of pain explored using the draw, write, and tell method. Journal for Specialists in Pediatric Nursing: JSPN, 23(3), e12218. 10.1111/jspn.12218 29790268

[R58] Pope N, Tallon M, Leslie G, & Wilson S (2019). Using ‘draw, write and tell’ to understand children’s health-related experiences. Nurse Researcher, 26(2), 42–45. 10.7748/nr.2018.e1594 30203931

[R59] Rafferty J (2018). Ensuring comprehensive care and support for transgender and gender-diverse children and adolescents. Pediatrics, 142(4), 14. 10.1542/peds.2018-2162 30224363

[R60] Rahilly EP (2015). The gender binary meets the gender-variant child: Parents’ negotiations with childhood gender variance. Gender & Society, 29(3), 338–361. 10.1177/0891243214563069

[R61] Riessman CK (1993). Narrative analysis. Sage Publications, Inc.

[R62] Rich AJ, Scheim AI, Koehoorn M, & Poteat T (2020). Non-HIV chronic disease burden among transgender populations globally: A systematic review and narrative synthesis. Preventive Medicine Reports, 20, 101259. 10.1016/j.pmedr.2020.101259 33335828 PMC7732872

[R63] Richardson L, & St. Pierre EA (2005). Writing: A method of inquiry. In Denzin NK & Lincoln YS (Eds.), The Sage handbook of qualitative research (3rd ed., pp. 959–978). SAGE.

[R64] Riley EA, Sitharthan G, Clemson L, & Diamond M (2011). The needs of gender-variant children and their parents: A parent survey. International Journal of Sexual Health, 23(3), 181–195. 10.1080/19317611.2011.593932

[R65] Robledo Castro C, Córdoba Andrade L, & Del Basto Sabogal LM (2023). Child-centered multimethod design: An approach to social representations in childhood education. Journal of Research in Childhood Education, 37(1), 1–19. 10.1080/02568543.2022.2048754

[R66] Schultz TR, Zoucha R, & Sekula LK (2022). The intersection between youth who identify as LGBTQ and emergency care for suicidality: An integrative review. Journal of Pediatric Nursing, 63, e82–e94. 10.1016/j.pedn.2021.10.008 34756491

[R67] Schutz A, & Luckmann T (1973). The Structures of the Life-World. Northwestern University Press.

[R68] Shires DA, Stroumsa D, Jaffee KD, & Woodford MR (2018). Primary Care Clinicians’ Willingness to Care for Transgender Patients. Annals of Family Medicine, 16(6), 555–558. 10.1370/afm.2298 30420373 PMC6231925

[R69] Steensma TD, Biemond R, de Boer F, & Cohen-Kettenis PT (2011). Desisting and persisting gender dysphoria after childhood: A qualitative follow-up study. Clinical Child Psychology and Psychiatry, 16(4), 499–516. 10.1177/1359104510378303 21216800

[R70] Steensma TD, Kreukels BP, de Vries AL, & Cohen-Kettenis PT (2013a). Gender identity development in adolescence. Hormones and Behavior, 64(2), 288–297. 10.1016/j.yhbeh.2013.02.0 23998673

[R71] Steensma TD, McGuire JK, Kreukels BP, Beekman AJ, & Cohen-Kettenis PT (2013b). Factors associated with desistence and persistence of childhood gender dysphoria: A quantitative follow-up study. Journal of the American Academy of Child and Adolescent Psychiatry, 52(6), 582–590. 10.1016/j.jaac.2013.03.016 23702447

[R72] Stryker S (1994). My words to Victor Frankenstein above the village of Chamounix: Performing transgender rage. GLQ: A Journal of Lesbian and Gay Studies, 1(3), 237–254. 10.1215/10642684-1-3-237

[R73] Stryker S (2006). (De)Subjugated knowledges: An introduction to transgender studies. In Stryker S & Whittle S (Eds.), The transgender studies reader (pp. 1–17). Routledge.

[R74] Te One S (2011). Supporting children’s participation rights: Curriculum and research approaches. In Hartcourt D, Perry B, & Waller T (Eds.), Researching Young Children’s Perspectives: Debarting the ethics and dilemmas of educational research with children (pp. 85–99). Taylor & Francis Group.

[R75] Tyler TR, Huddleston BS, Kronner HW, Calloway ET, Martin KG, Morgan AL, Aguilar R, Wheeler SA, Barnett FA, Kohring CL, Spaeth CM, Abbott KG, Montgomery KM, Preston M, Barton TN, Thornton MH, Munoz V, & Dean DE (2025). Transgender and Gender-Diverse Children’s Health Care Experiences With Parents. Families in Society: The Journal of Contemporary Social Services, 2025(0), 623. 10.1177/10443894251314623

[R76] Van Manen M (1997). Researching lived experience: Human science for an action sensitive pedagogy. The Althouse Press.

[R77] Warner M (1991). Introduction: Fear of a Queer Planet. Social Text, 29, 3–17.

[R78] Water T, Payam S, Tokolahi E, Reay S, & Wrapson J (2020). Ethical and practical challenges of conducting art-based research with children/young people in the public space of a children’s outpatient department. Journal of Child Health Care: For Professionals Working with Children in the Hospital and Community, 24(1), 33–45. 10.1177/1367493518807318 30376719

[R79] Weedon C (1987). Feminist Practice and Poststructuralist Theory. Blackwell.

[R80] Weiselberg EC, Shadianloo S, & Fisher M (2019). Overview of care for transgender children and youth. Current Problems in Pediatric and Adolescent Health Care, 49(9), 100682. 10.1016/j.cppeds.2019.100682 31706835

[R81] Wiseman N, Rossmann C, Lee J, & Harris N (2019). “It’s like you are in the jungle”: Using the draw-and- tell method to explore preschool children’s play preferences and factors that shape their active play. Health Promotion Journal of Australia: Official Journal of Australian Association of Health Promotion Professionals, 30 Suppl 1(S1), 85–94. 10.1002/hpja.209 30267607

[R82] Zaman BA, & Anderson-Nathe B (2021). Toward queer potentialities in child and youth care. International Journal of Child, Youth and Family Studies, 12(3–4), 104–128. 10.18357/ijcyfs123-4202120341

